# Is it safe for pregnant health-care professionals to handle cytotoxic drugs? A review of the literature and recommendations

**DOI:** 10.3332/ecancer.2014.418

**Published:** 2014-04-10

**Authors:** S Gilani, S Giridharan

**Affiliations:** University Hospital of North Staffordshire, ST4 6QG, UK

**Keywords:** chemotherapy, pregnant, nurse, health-care professional

## Abstract

The information related to health risks to foetuses due to the handling of chemotherapeutic agents by nurses during pregnancy is limited. The risks involved can be reduced significantly if nurses adhere to standard safety precautions while handling cytotoxic drugs. Nurses in patient areas where chemotherapy is administered are at constant low-level risk of exposure. The authors tried to gather evidence in this article from the recent literature to help to formalise policies for pregnant mothers working in these settings.

## Introduction

How safe is it for pregnant health-care professionals to handle chemotherapy drugs? The answer to this question is not straightforward. We attempted to look at the current literature to assimilate the evidence involved with answering this important question.

Chemotherapy services have expanded over recent years. Mothers remain concerned about the safety of their unborn babies while handling hazardous materials. Most chemotherapy drugs are cytotoxic. This means they may be mutagenic, carcinogenic, or teratogenic in nature. How toxic they can be during pregnancy is not absolutely clear. Therefore, handling such drugs may not be 100% safe in pregnancy.

Unfortunately, the information is scanty. The handling of these chemicals by pregnant nurses may be risky. However, the risk may be negligible if one adheres to standard safety precautions [1]. That is why most governing bodies have issued guidelines. For example, the Oncology Nursing Society UK says, ‘the risks cannot be completely eliminated even after adhering to all precautions. Therefore, an additional level of protection is suggested for those most vulnerable to reproductive and development effects of hazardous drugs’ [2]. Similarly, the American Society of Health System Pharmacists says, ‘Personnel may be exposed to hazardous drugs at many points during manufacture, transport, distribution, storage, and administration, as well as during waste handling and equipment maintenance. All workers involved in these activities have potential of contact with uncontained drug’ [3]. In the same way, the National Institute for Occupational Safety and Health (NIOSH) has stated that ‘…measured detectable concentration of one to five hazardous drugs in various locations such as Biological Safety Cabinet (BSC) surfaces, floors, countertops, storage areas, tables, and chairs in patient treatment areas and locations adjacent to drug handling areas…’. There is a possibility of at least skin exposure in this environment. Therefore, people who handle chemotherapeutic drugs are at constant risk of exposure. This may be true for nurses who administer chemotherapy. This risk of exposure can be minimised by personal protective equipment (PPE), a needle-less system, and ventilation cabinets. However, this may not eliminate all possible risks [4].

## Adverse effects during pregnancy

Chemotherapy drugs have adverse effects mostly on rapidly dividing cells. Exposure to such chemicals can lead to growth abnormalities in the foetus. Moreover, a high metabolic rate in the foetus leads to significant exposure if the mother is exposed through skin, inhalation, or ingestion [5].

Pregnancy has three phases: embryogenesis, organogenesis, and foetal growth. The effects on the foetus are different during each phase. Embryogenesis kicks in at fertilisation and ends on the 14th day. Cell damage in this phase usually leads to foetal death. During organogenesis, the foetus forms different organs and body systems. This lasts to the end of the 12th week. Damage can result in spontaneous abortion or malformation. The last phase starts at the 12th week and ends at birth. Functional abnormalities may result during this phase, manifesting as physiological or intellectual abnormalities [1].

## Wider context

The rapid expansion of chemotherapy services throughout the world has raised serious concerns regarding exposure by pregnant mothers to cytotoxic drugs. Much of the information is patchy. A recent study, published in the *American Journal of Obstetrics and Gynecology *[6], has looked at 7500 pregnant nurses involved in the handling of hazardous materials. It has shown a relative increase in rates of spontaneous abortion among mothers who handle chemotherapy. They did use PPE and were not involved in obvious exposure. This means that they were unknowingly exposed to constant low levels of exposure during their pregnancy [6]. This study has brought the working conditions of oncology nurses into the limelight. A recent article in the *Seattle Times* raises similar concerns [7].

Historically, nurses were found to be less adherent to guidelines while handling chemotherapy drugs. Scientific evidence is seriously lacking in this field [8]. A recent study among Greek oncology nurses showed a lack of adequate use of PPE during pregnancy [9]. Similarly, another study among Turkish Oncology Nurses highlighted a lack of protection during pregnancy with widespread non-adherence to standards set by national and international labour organisations [10].

## Safe handling of chemotherapy drugs

A safe working environment is essential for pregnant mothers. General guidelines are available, but they are not completely sufficient to protect unborn babies. Individuals and society are both responsible for maintaining the health of future generations. Every staff member has the responsibility for her own safety. Guidelines from the Health and Safety Executive UK for new and expectant mothers emphasise: ‘a safe level of exposure cannot be determined for cytotoxic drugs, so individuals should avoid exposure or reduce it to as low a level as reasonably practicable’ [12]. One should keep reviewing safe working conditions constantly. The Oncology Nursing Society (ONS) recommends to hospitals, ‘Alternate duty which does not include administration or handling of cytotoxic agents must be made available to both men and women involved in planning a pregnancy when required’ [2]. Breast feeding or pregnant nursing staff who wear PPE should consider themselves safe even while administrating chemotherapy, cleaning up bodily fluids, or handling pre-mixed drugs. The use of PPE has been shown to reduce the risk of exposure significantly [3].

However, the ONS also states that one remains at risk of exposure even while wearing PPE. Studies have shown drugs being excreted into breast milk. They recommended that lactating mothers not handle these drugs [13]. Similarly, the American society of Heath-System Pharmacists has feared environmental contamination by these drugs from BSCs onto the gloves, the final product, or into the air [3].

In another study, two infants who were fed on breast milk from mothers exposed to cyclophasphamide were found to be neutropenic [14]. Ethically, it is not possible to run studies to see the effects of exposure of drugs on infants of lactating mothers. According to the American Academy of Paediatrics cytotoxic drugs in the breast milk were found to interfere with cellular metabolism in infants [15]. These drugs can cause bone marrow suppression including neutropenia and growth retardation. Therefore, exclusion of these drugs from s pregnant mother’s environment is important. According to the Hazardous Waste Regulations 2005 [16], different cytotoxic drugs may have one or more adverse effects on living cells. They may be carcinogenic (H7), genotoxic (H6), teratogenic (H10), or mutagenic (H11) in nature. This information is normally written on the material safety data sheet provided with the drug. Based on this information, nurses involved in handling them should take extra precautions to reduce risks posed to them and their babies [1].

Most nurses consider using PPE inconvenient. Due to this, many do not utilise the facility. Nurses are relatively at higher risk of adverse effects from cytotoxic drugs than their patients due to constant exposure. In 2009, these effects were discussed in the ONS meeting in detail [17]. According to ONS, these drugs have effects on the reproductive system leading to menstrual dysfunction, infertility, miscarriage, foetal abnormalities, and premature labour. They can also lead to mental and physical disabilities in children. ONS stated, ‘Multiple studies have reported an increase in malignancies in nurses, physicians, and pharmacists who handled hazardous drugs [18]. In 2006, a study showed 75% to 100% of vials of cyclophosphamide, 5Fu and doxorubicin were already contaminated before their arrival in the centre. Guidelines from ONS, NIOSH, and other institutions suggest using BSC and PPE. These devices should have external ventilation. PPE should also include a face fitting mask, two layers of chemotherapy approved gloves and impermeable gowns [1]. Contamination of floors, table-tops, phones, chairs, and devices are a common source of unrecognised hazard. This type of surface contamination is the most common form of contamination [19].

## Conclusion

Long-term studies are needed to evaluate the effects of low-level exposure to cytotoxic drugs by pregnant and lactating mothers. General guidelines are available [12]. Especially, during the first trimester, exposure should be limited as much as possible. During the second and third trimesters, work may be allowed if standard safety precautions are followed [21]. In brief, the responsibility lies with nurses to follow the guidelines [22]. At the very first indication of suspected exposure, they should try to remove themselves from the source [1].

## Recommendations based on current evidence

The following recommendations are made to avoid exposure to foetuses and infants:

1. All staff involved in the handling and administration of cytotoxic drugs should be familiar with and adhere to local and national policies, and follow safe practice with cytotoxic drugs following standard operating procedures.
2. Pregnant staff should be given the choice to avoid work activity in an area with increased risk of exposure to chemotherapy agents.
3. It is the responsibility of the nursing staff to inform the employer regarding their decision to conceive, when they become pregnant, or when they lactate.
4. Nursing staff should avoid working in high-risk areas during the first 84 days of their pregnancy.
5. After 84 days of pregnancy, nursing staff can work in these areas if they adhere to standard precautions using PPE.
6. Lactating mothers should also avoid working in high-risk chemotherapy areas.
7. All staff involved in handling and administering chemotherapy must undergo appropriate training and education for safe handling of cytotoxic drugs.
8. Employers should take responsibility to facilitate avoiding exposure and provide local guidelines.

## References

[1] Albin K (2010). Administering chemotherapy: is it safe for pregnant or breast-feeding veterinary technicians?. Vet Tech.

[2] Polovich M, Whitford JM, Olsen M (2009). Chemotherapy and Biotherapy Guidelines and Recommendations for Practice.

[3] (2004). Preventing Occupational Exposure to Antineoplastic and Other Hazardous Drugs in Health Care Settings.

[4] American Society of Health-System Pharmacists (2006). ASHP guidelines on handling hazardous drugs. Am J Health-Syst Pharm.

[5] Paskulin GA (2005). Combined chemotherapy and teratogenicity. Birth Defects Res A Clin Mol Teratol.

[6] Christiana C Lawson (2012). Occupational exposures among nurses and risk of spontaneous abortion. Am J Obst Gynaecol.

[7] Smith C (2010). Life saving drugs may be killing health care workers. Seattle Times.

[8] Martin S (2003). Chemotherapy-handling practices of outpatient and office-based oncology nurses. Oncol Nurs Forum.

[9] Constantinidis T (2011). Occupational health and safety of personnel handling chemotherapeutic agents in Greek hospitals. Eur J Cancer Care.

[10] Baykal U, Seren S, Sokmen S (2009). A description of oncology nurse’s working conditions in Turkey. Eur J Oncol Nurs.

[11] OSHA (1999). Osha Technical Manual, Ted 1-0 15a Section VI. Controlling occupational exposure to hazardous drugs.

[12] (2002). New and expectant mothers at work: a guidelines for employers. Health Saf Exec.

[13] Hogle WP (2004). Chemotherapy safety. Oncol Nurs Soc Conf.

[14] Durodola JI (1979). Administration of cyclophosphamide during late pregnancy and early lactation: a case report. J Natl Med Assoc.

[15] American Academy of Pediatrics (2001). The transfer of drugs and other chemicals into human milk. J Am Acad Pediatr.

[16] Hazordous Waste Regulations (2005). http://www.legislation.gov.uk/uksi/2005/894/contents/made.

[17] Connor TH, McDiarmid MA (2006). Preventing occupational exposures to antineoplastic drugs in health care settings. CA Cancer J Clin.

[18] (2009). Nurse safety with hazardous drugs: where do you stand?. J Oncol Nurs Soc.

[19] Fransman W (2007). Nurses with dermal exposure to antineoplastic drugs. J Epidemiol.

[20] (2002). New and expectant mothers at work: a guidelines for employers. Health Saf Exec.

[21] (2003). Safe handling of cytotoxic drugs. Health Saf Exec.

[22] (2007). MARCH Guidelines: “Pregnancy in Staff Handling Cytotoxics”.

